# Is dangling of the lower leg after a free flap reconstruction necessary? Study protocol for a large multicenter randomized controlled study

**DOI:** 10.1186/s13063-019-3665-0

**Published:** 2019-09-11

**Authors:** David D. Krijgh, Teun Teunis, Pascal P. A. Schellekens, Marc A. M. Mureau, Antonius J. M. Luijsterburg, Tallechien M. T. Tempelman, Eva S. J. van der Beek, Wiesje Maarse, J. Henk Coert

**Affiliations:** 1 Department of Plastic and Reconstructive Surgery, University Medical Center Utrecht, Heidelberglaan 100, 3508 GA Utrecht, Postbus 85500, the Netherlands; 2 Department of Plastic and Reconstructive Surgery, Erasmus MC, University Medical Center Rotterdam, Rotterdam, the Netherlands; 3 Department of Plastic and Reconstructive Surgery, University Medical Center Groningen, Groningen, the Netherlands

**Keywords:** Free flap, Lower leg, Dangling, Complications, Physical function, Infection, Union, Costs

## Abstract

**Background:**

Within the field of plastic surgery, free tissue transfer is common practice for knee and lower leg defects. Usually, after such free flap reconstruction, patients undergo a dangling protocol in the postoperative phase. A dangling protocol is designed to gradually subject the free flap to increased venous pressure resulting from gravitational forces. Worldwide there are multiple variations of dangling protocols. However, there is no evidence available in the literature that supports the use of a dangling protocol.

**Methods:**

This is a multicenter randomized controlled trial that includes patients with a free flap lower leg reconstruction. The primary outcome is to assess whether a no-dangling protocol is not inferior to a dangling protocol, in terms of proportion of partial flap loss, 6 months after surgery. Secondary objectives are to identify differences in major and minor complications, length of stay, and costs, and to objectify blood gaseous changes during dangling. Furthermore, at 2 years we will assess difference in physical function, infection rates, and osseous union rates.

**Discussion:**

The primary outcome of this study will give a more decisive answer to the question of whether a dangling protocol is necessary after a free flap reconstruction of the lower leg. The secondary outcomes of this study will provide a better insight into the physical functions, infection rates, and union rates in these patients.

**Trial registration:**

Central Committee on Research Involving Human Subjects (CCMO), NL63146.041.17. Registered on 11 July 2018. Netherlands Trial Register, NTR7545. Registered on 10 October 2018.

**Electronic supplementary material:**

The online version of this article (10.1186/s13063-019-3665-0) contains supplementary material, which is available to authorized users.

## Background

Within the field of plastic surgery, free tissue transfer is common practice. In knee and lower leg defects due to trauma, oncological resection, or chronic infection, adequate soft tissue coverage of bony structures is imminent. In case of insufficient bony coverage, a muscle or fasciocutaneous (skin, fat, and fascia) free tissue transplantation is performed. This is a microsurgical operation in which distant body tissue is transplanted to the defect. However, there is great diversity in the postoperative care for patients with a lower leg reconstruction. Usually, after such free flap reconstruction, patients undergo a dangling protocol in the postoperative phase. During this dangling protocol, the patients hang the reconstructed lower leg from the side of the bed in order to gradually subject the free flap to increased venous pressure resulting from gravitational forces. Worldwide there are multiple variations of dangling protocols. The starting point, frequency, and duration vary widely; whereas some start the dangling protocol as early as on the second postoperative day (POD), others wait until the fourth postoperative week [1–3]. Some report not to use dangling as a standard procedure at all [1], and some use it only in select cases [2]. In general, the dangling protocol is performed in a hospital setting. Although not supported by any evidence, dangling protocols are designed to decrease the risk of postoperative complications such as partial or total flap necrosis. These protocols usually extend hospital stay, resulting in higher costs.

Jokuszies et al. [4] and Neubert et al. [5] performed the only two known randomized controlled trials in this patient group. They compared an early start of the dangling protocol on POD 3 to the more standard starting point on POD 7. Both studies showed that the combined wrapping and dangling procedure can safely be started at POD 3. It must be noted that the patients included in these studies were for the most part the same group of patients. Furthermore, the numbers of patients in both studies were small (31 and 49), resulting in an underpowered study [4, 5]. McGhee et al. published a systematic review comparing an early dangling protocol to a late dangling protocol [6]. They found eight relevant articles, including the studies performed by Jokuszies et al. [4] and Neubert et al. [5]. Based on the currently available literature, they concluded that an early dangling protocol can be safely used. A more recent systematic review by Soteropulos et al. came to a similar conclusion [7].

Both systematic reviews are calling for a larger randomized controlled trial. Moreover, whether or not to dangle at all is an intriguing question. We hypothesize that the free flap as a result of flap ingrowth regains its venoarterial response and will develop increased venous outflow over time. We believe that this is independent of a dangling protocol. Therefore, we designed a study protocol for a large multicenter randomized controlled trial, which we present in this article.

### Objectives

The primary objective is to assess whether a no-dangling protocol is not inferior to a dangling protocol. Our primary outcome is measured in terms of proportion of patients who experienced partial flap loss which did not require another free flap procedure. Total follow-up for our primary objective is 6 months. Based on a rate of partial flap loss of 6%, we decided that an absolute increase in incidence of 12% would be clinically significant. We will calculate the absolute risk differences in incidence of partial flap loss (major and minor combined) between groups.

We also have the following secondary objectives:

- We hypothesize that there is no difference in one or more major complications at 6 months.
- We would like to objectify the gaseous changes within the free flap during the dangling protocol in a select group of patients.
- We will measure the physical functions at 3 and 6 months and at 1, 1.5, and 2 years with the Patient-Reported Outcomes Measurement Information System (PROMIS) physical function, EuroQol five dimensions (EQ-5D), and visual analog scale (VAS) questionnaires.
- We hypothesize that there is no difference in the number of patients experiencing one or more minor complications at 3 and 6 months postoperatively.
- We will investigate whether there is a difference in the length of hospital stay between the two groups and perform a costs analysis.
- We will investigate infection rates and osseous union rates with a follow-up of 2 years.

## Methods/design

### Trial design

**Fig. 1:**
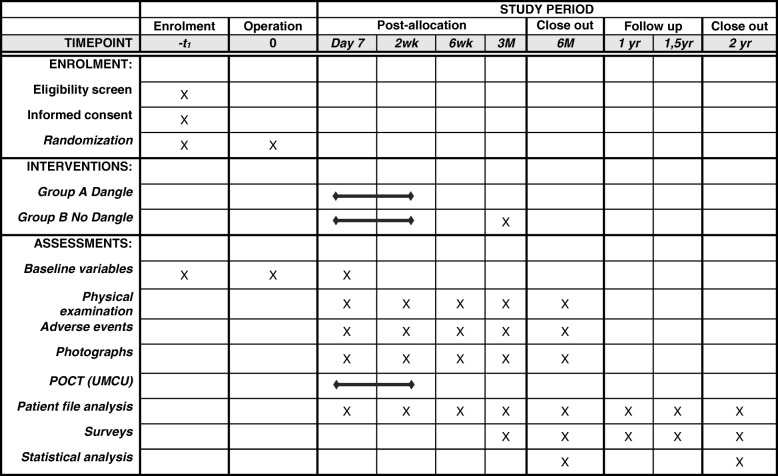
Standard Protocol Items: Recommendations for Interventional Trials (SPIRIT) figure showing the phases of the trial and data collection time points

The study is taking place at three Dutch hospitals: University Medical Center (UMC) Utrecht, Erasmus University Medical Center (Erasmus MC) Rotterdam, and UMC Groningen. A dangling protocol is the current standard of care in these hospitals. Patients are randomly assigned by a computer program to group A or B. Patients in group A start a dangling protocol on POD 7. Patients in group B can mobilize without limitations starting on POD 7 and are discharged from the hospital when possible. All patients will be seen on POD 12–15, after 5–7 weeks, 2.5–3.5 months, and 5–7 months at the ward or the outpatient clinic. During these visits the complications will be evaluated. The study phases and data collection time points are shown in Fig. 1. The Standard Protocol Items: Recommendations for Interventional Trials (SPIRIT) checklist is provided as Additional file 1.

Patients in group A (dangling) at the UMC Utrecht will undergo blood tests with the use of a point-of-care testing (POCT) device. A drop of blood will be taken on a daily basis from both the flap and from the contralateral leg (control) and will subsequently be analyzed for pO2, pCO2, and pH levels. Moreover, all patients will be asked to fill out three short (PROMIS, EQ-5D, and VAS) online questionnaires at 3 and 6 months and at 1, 1.5, and 2 years postoperative. The inclusion and exclusion criteria are listed in Table 1.

**Table 1:** Inclusion and exclusion criteria for “the dangle study”

Inclusion criteria	- Male or female
	- Age between 18 and 99 years old
	- Lower leg defect in need of a free flap reconstruction
Exclusion criteria	- Age under 18 years
	- Co-morbidities that prevent the patient from being able to undergo a dangling protocol
	- Insufficient Dutch language skills to understand the study
	- Patients who are mentally incompetent or are unable to give informed consent
	- Reconstruction with 2 or more free flaps
	- Patients who are getting a secondary free flap due to partial or total free flap necrosis

### Study parameters

As our primary study parameter we will investigate whether there is a significant increase in the incidence of partial flap loss in patients who did not undergo a dangling protocol (group B) versus patients who did undergo a dangling protocol (group A). We defined complete flap necrosis, partial flap necrosis (if a revision surgery with a second free flap is necessary), and pulmonary embolism as major complications. Screening for pulmonary embolism will only be performed if there is a clinical suspicion of a pulmonary embolism. Partial flap loss is defined as a minor complication if no secondary free flap is needed or as a major complication if a secondary free flap was needed. Wound dehiscence, wound infection, failure of skin graft ingrowth on the free flap, and hematoma for which a surgical exploration was needed were defined as minor complications.

### Randomization

Randomization will be performed in Castor EDC, Amsterdam, the Netherlands. This study cannot be blinded. The randomization is stratified per medical center. The coordinating researcher will log in to the computer system and fill out the required information. In case of a re-intervention, the patient will still be included in the study. If the arterial and/or venous anastomosis required a redo, then the day of the performed re-intervention will be POD 0. The study (dangling or no-dangling protocol) will start 7 days after the re-intervention.

In case the patient has a partial or complete flap loss and a secondary free flap transplantation is indicated, the patient will not be re-invited to join the study for randomization after the secondary free flap.

### Study procedures

All patients randomized to group A will undergo a dangling protocol. In Table 2 this dangling protocol is further specified. The flap will be wrapped during the dangling procedure. After 5–7 weeks, 2.5–3.5 months, and 5–7 months, the patients will be seen at the outpatient clinic to evaluate complications. Photographs of the flap will be taken on POD 6 or 7 and at all planned follow-up visits. These photos will be used in a sample test to check whether the estimated percentage partial flap necrosis and skin graft take was estimated correctly.

**Table 2:** Dangling protocol for patients in group A in the free flap group

Postoperative day	Duration of dangling 3 times a day
7	5 min
8	10 min
9	20 min
10	30 min
11	45 min
12	60 min
13	Unlimited

Patients in group B are allowed to dangle for an unlimited time starting on POD 7. Patients in group B are allowed to go home (if the wound status and further co-morbidities allow) and will be seen at the outpatient office between PODs 12 and 15 (depending on the weekends) and after 5–7 weeks, 2.5–3.5 months, and 5–7 months. From POD 7 until POD 14 patients will wear a step counter and will keep track of the time they spent standing or sitting with their leg down. The flap will be wrapped starting on POD 7. During the outpatient office visits the complications will be evaluated.

### Point-of-care blood tests

All of the randomized patients in group A at the UMC Utrecht will undergo blood tests. Using a POCT device, a drop of blood from the free flap and from the contralateral “healthy” leg (control group) will be analyzed for blood gases (pO2, pCO2, pH). If the patient has a bilateral leg injury, the drop of blood will be taken from a different body part. This study is designed to get a better insight into the gaseous changes within the free flap during the dangling process. Blood will be taken from the free flap and the contralateral leg at the beginning and at the end of the dangling session. Table 3 illustrates the protocol for the POCT. The goal of these POCT measurements is to present a curve of the gaseous changes during a dangling protocol and to get more insight into the effect of dangling on these blood gases.

**Table 3:** Point-of-care testing (POCT) protocol during the dangling protocol

Postoperative day	Before a dangling session	At the end of a dangling session
7	POCT from flap and contralateral leg	POCT from flap
8	POCT from flap and contralateral leg	POCT from flap
9	POCT from flap and contralateral leg	POCT from flap
10	POCT from flap and contralateral leg	POCT from flap
11	POCT from flap and contralateral leg	POCT from flap
12	POCT from flap and contralateral leg	POCT from flap

### Anticoagulant use during the study

The use of coumarins or a novel oral anticoagulant will be preoperatively stopped. If the patient has an indication for “bridging” with a therapeutic dose of low molecular weight heparin (LMWH) due to co-morbidities, this will be done. If bridging is not indicated, the patient will receive a prophylactic dose of LMWH to reduce the risk of deep venous thrombosis or pulmonary embolism, which is standard of care for all patients undergoing surgery in the Netherlands.

Patients who use acetylsalicylic acid will continue this medication. Acetylsalicylic acid will not be started in patients after a free flap, in case the patients did not use this medication before the operation.

### Sample size calculation

Based on the incidence of partial flap necrosis in the meta-analysis by Xiong et al., we performed a power calculation [8]. Because total free flap loss rarely occurs after POD 5, we decided to focus in this study on partial flap loss. Based on a rate of partial flap loss of 6%, we decided that an absolute increase in incidence of 12% would be clinically significant. To detect this non-inferiority margin with 80% power and a two-sided 95% confidence interval (CI) and 5% estimated loss to follow-up, we aim to include 130 patients. Each year about a hundred lower limb reconstructions are performed at our three hospitals, resulting in an inclusion time of 2 years.

### Statistical analysis

As the primary hypothesis we will calculate the absolute risk differences in incidence of partial flap loss (major and minor combined) between groups. If the upper limit of the 95% CI falls within 12%, we regard this as a non-inferiority difference.

The secondary hypotheses will be tested for superiority: absolute risk difference for one or more major complications and 95% CI, and linear regression for length of stay, physical function, and blood tests.

We will perform an intention-to-treat analysis. However, since investigators can decide to withdraw a subject from the study for life-threatening medical reasons (see the subsequent section on Recruitment, consent, and withdrawal), we will perform an additional per protocol analysis and compare baseline characteristics of patients withdrawn and patients lost to follow-up. We will use multiple imputation to account for missing variables.

We will perform an interim analysis when 40 patients are randomized. If we find a significant difference through Fisher’s exact test in patients having one or more major or minor complications, we will terminate the trial. Based on previous studies, we will test our final non-inferiority hypothesis without alpha adjustment for the superiority tested interim analysis.

### Recruitment, consent, and withdrawal

Patients will be invited by a plastic surgery resident who is not affiliated with this study or clinically responsible for the patient. This will be done before POD 5. The patient will then have at least 24 h to decide whether he/she would like to join the study. The patient will be provided with an information letter and asked to give written informed consent. The study will start on POD 7. If a patient at the UMC Utrecht is randomized to group A and would like to take part in the study but does not want to have the POCT performed, then the patient will still remain in group A (without the blood test).

Based on the current literature, there are no known increased risks involved with participation in this study. The hypothesized beneficial effect is that patients in group B might have a shorter hospital stay.

Subjects can leave the study at any time for any reason if they wish to do so, without any consequences. The investigator can decide to withdraw a subject from the study for life-threatening medical reasons. If during the study it is anticipated that there is a very high risk of total flap necrosis, the treating physician can decide to withdraw the patient from the dangling part of the study. This applies to patients in both groups A and B. This risk will be an estimation based on the clinical experience of the surgeon. For ethical reasons, we believe that this possibility is important in this study. The patient will still be included for the secondary objectives. If a patient experiences an adverse event which prevents the patient from adequately performing a dangling or a non-dangling protocol, that patient will be terminated from the study for the primary study outcome. If possible, the patient will still be included for the secondary objectives.

### Study data management, oversight, and publication

Data will be handled confidentially. Data will be collected in the electronic patient file by the local researcher and subsequently registered in Castor EDC, Amsterdam, the Netherlands. Patients will be anonymized. The handling of personal data will be performed in compliance with the Dutch Personal Data Protection Act and in compliance with Good Clinical Practice guidelines. Data will be kept for 15 years. Informed consent from study participants will be recorded at every hospital in the electronic patient file, and signed paper forms will be securely locked away within the hospital where the patient is undergoing treatment.

The study is monitored by an independent monitoring company (Julius Clinical, Zeist, the Netherlands) according to a detailed monitoring plan. Insurance is provided for all participants in accordance with Dutch legislation. The results of this study will be submitted to peer-reviewed journals.

## Discussion

This study is designed to give a more decisive answer to the question of whether a dangling protocol is necessary after a free flap reconstruction of the lower leg. This will be the first randomized controlled study comparing a non-dangling protocol to a dangling protocol. Worldwide, the current standard of care is a dangling protocol. We believe that the length of hospitalization in a non-dangling protocol can be significantly reduced compared to that for a dangling protocol. This would potentially reduce infection rates and lower costs. Furthermore, patients undergoing a non-dangling protocol will be able to ambulate at an earlier stage, reducing the risk of deep vein thrombosis and pulmonary embolism.

Our POCT measurements are a novel way to provide us with detailed information about the gaseous changes in a free flap during dangling. Furthermore, we will be able to give more insight into the physical functions, infection rates, and osseous union rates for this patient group.

At the end of this study we will have included 130 patients. This will be the largest prospective study in this patient group. Therefore, we will also be able to collect unique data about the physical functions, infection rates, and union rates.

### Limitations

Given that this is a non-blinded study, patients who are randomized to the no-dangling group could implement a dangling protocol on their own. To prevent this, we will provide patients in this group with a step counter, and they will have to keep track of the amount of time that their leg was in a dangling position.

For ethical reasons, we believe that it is important that the treating physician can decide to withdraw a patient from the dangling part of the study if the physician thinks the risk of flap necrosis is too high. However, this could lead to treatment indication bias. However, we should be able to account for this by intention-to-treat or per protocol analysis.

Our sample size calculation is based on a rate of partial flap loss of 6%. We decided that a rather high absolute increase in incidence of 12% would be clinically significant. If we had chosen a lower increase of incidence the sample size would have increased significantly, resulting in a non-feasible study.

### Trial status

The study was registered at the CCMO (Central Committee on Research Involving Human Subjects) in the Netherlands on 11 July 2018 (NL63146.041.17) and registered with the Netherlands Trial Register (registration number NTR7545). This article is based on protocol version number 7.0, dated 22 January 2019. Recruitment started at the UMC Utrecht on 16 October 2018, at the Erasmus MC Rotterdam on 17 January 2019, and at the UMC Groningen on 29 January 2019. The approximate date on which recruitment will be completed is 1 July 2021.

## Additional file

Additional file 1: Spirit flowchart for The Dangle Study. (DOC 122 kb)

## Data Availability

Data sharing is not applicable to this article, as no datasets will be generated or analyzed during the current study.

## Abbreviations

CCMO: Centrale Commissie Mensgebonden Onderzoek
EQ-5D: EuroQol five dimensions
POCT: Point-of-care testing
POD: Postoperative day
PROMIS: Patient-Reported Outcomes Measurement Information System
UMC: University Medical Center
VAS: Visual analog scale
